# Total inotrope exposure score: an extension of the vasoactive inotrope score

**DOI:** 10.1186/2197-425X-3-S1-A211

**Published:** 2015-10-01

**Authors:** H Bangalore, P Checchia, E Ocampo, J Heinle, D Guffey, C Minard, M Gaies, LS Shekerdemian

**Affiliations:** Great Ormond Street Hospital, Paediatric Intensive Care Unit, London, United Kingdom; Texas Childrens Hospital, Pediatric Critical Care, Houston, TX United States; Texas Childrens Hospital, Pediatric Cardiology, Houston, TX United States; Texas Childrens Hospital, Pediatric Congenital Heart Surgery, Houston, TX United States; Baylor College of Medicine, Translational Research and Biostatistics, Houston, TX United States; CS Mott Children's Hospital, Pediatric Cardiology, Ann Arbor, MI United States

## Intr

To incorporate advances in documentation with computerised Electronic Medical Record systems, enable ease of calculation, and provide a single score for vasoactive support after cardiac surgery in children across all ages, we explore a modification of the VIS, which is the Total Inotrope Exposure Score (TIES).

## Objectives

To study the performance of the TIES as compared to the VIS.

## Methods

A single-centred retrospective study was conducted in children undergoing cardiac surgery with Cardiopulmonary bypass (CBP) between September 2010 and May 2011. The average and maximum VIS at 24 and 48 hours (VIS_avg_24, VIS_max_24, VIS_max_48, VIS_avg_48) and TIES were calculated. The performance of these scores to predict primary clinical outcome-- either death, Cardiopulmonary resuscitation, extra-corporeal membrane oxygenation (ECMO) before hospital discharge and secondary outcomes—prolonged length of invasive mechanical ventilation, length of intensive care unit(ICU) stay and hospital stay were calculated.

## Results

167 separate admissions included 37 (22.2%) neonates and 65 (41.3%) infants with a mean age of 2.9 (6.0) years. Twenty percent had higher complexity operations with a RACHS-1 category of 4-6.

The TIES predicted the primary outcome (6 of 167 cases) with an AUC 0.92 (0.86, 0.97). The unadjusted odds ratio for a poor outcome was OR 42 (4.8, 369.6)].

The TIES score best predicted prolonged ventilation with an AUC of 0.88 (0.81, 0.95) compared to VIS_avg_24 - 0.73 (0.64, 0.82) [p = 0.0001], VIS_max_24 - 0.74 (0.66, 0.83) [p = 0.0011], VIS_avg_48 - 0.74

(0.65, 0.82) [p < 0.0001], VIS_max_48 - 0.67 (0.58, 0.77) [p < 0.001]. TIES was the best predictor of prolonged mechanical ventilation with an adjusted OR of 13.6 (3.9, 47.8) at a cut off of 14.7. None of the other scores significantly predicted prolonged ventilation after adjusting for covariates in a multiple regression. The TIES score best predicted freedom from mechanical ventilation with an adjusted-hazard ratio of Cox proportional hazard model of 0.97 (0.96, 0.99) [p = 0.01].

Similarly, the adjusted odds of a prolonged CVICU stay was predicted by the TIES score with an OR of *≥*12.7 (3.51, 45.7) at a cut off of 14.7. The other scores which predicted a prolonged CVICU stay were - VIS_avg_24 ≥4.5 OR 4.3(1.3, 14.1); VIS_max_24 ≥4.8 OR 7.3(2.2, 24.2); VISavg48 ≥3.1 OR 3.5(1.1, 10.7).

The odds of a prolonged hospital stay were 4 (1.37, 11.66) and 12.2 (2.5, 60.9) for VIS_avg_48 and TIES at cut offs of 3.1 and 14.7 respectively. The TIES score best predicted discharge from the ICU and hospital with an adjusted Hazard Ratio of 0.96(0.94, 0.98)[p < 0.001] and 0.98(0.96,0.99)[p < 0.024].

## Conclusions

The TIES score performed well and warrants prospective validation across larger numbers of patients, and across institutions.

